# Undernutrition and Intestinal Infections in Children: A Narrative Review

**DOI:** 10.3390/nu17091479

**Published:** 2025-04-28

**Authors:** Maria Clara da Cruz Carvalho, Samilly Albuquerque Ribeiro, Lélia Sales de Sousa, Aldo Ângelo Moreira Lima, Bruna Leal Lima Maciel

**Affiliations:** 1 Graduate Program in Health Science, Center for Health Science, Federal University of Rio Grande do Norte, Natal 59078-970, RN, Brazil; maria.claracc@hotmail.com; 2 National Institute of Biomedicine of the Brazilian SemiArid, Faculty of Medicine, Federal University of Ceara, Fortaleza 60430-275, CE, Brazil; samilly.rib@gmail.com (S.A.R.); alima@ufc.br (A.Â.M.L.); 3 Christus University Center, Fortaleza 60160-230, CE, Brazil; lelia.doc@alu.ufc.br; 4 Department of Nutrition, Center for Health Science, Federal University of Rio Grande do Norte, Natal 59078-970, RN, Brazil

**Keywords:** enteropathy, infants, enteric infections, intestinal barrier function

## Abstract

Undernutrition affects thousands of children under five years old worldwide, and various factors are related to its onset, among which we highlight enteric infections and gastrointestinal barrier dysfunction. The cycle of intestinal infections and undernutrition has long-term consequences, such as cognitive deficits, poor growth, and metabolic diseases in adulthood. This review explores factors linked to childhood undernutrition, focusing on intestinal infections and markers of intestinal permeability that affect child development. This narrative review was conducted using Medline/PubMed, Web of Science, and Scopus, from July 2024 to March 2025. Studies involving children under five years old and addressing undernutrition, intestinal infections, or intestinal permeability markers were included. Exclusion criteria comprised studies without therapeutic focus, and books, case reports, or academic theses. No language restrictions were applied, and registration on global platforms was not required. Overall, the studies reported a close relationship between enteric pathogens, diarrheal and non-diarrheal stools, and undernutrition. Among the pathogens most frequently found in the feces of malnourished children were *Shigella*, enterotoxigenic *Escherichia coli*, enteroaggregative *E. coli* (EAEC), and *Cryptosporidium*. The studies also showed the relationship between gastrointestinal barrier function and undernutrition, with the deterioration of nutrient absorption and, consequently, repercussions on development, linear growth, and weight in children. Although the studies analyzed had different designs and heterogeneity in the age range of the studied children, it was possible to observe the relationship between the infection/undernutrition cycle. Future studies should optimize personalized nutrient-based therapies, assess long-term effects on gut health and growth, and explore the gut microbiome’s role in enteric infection susceptibility and undernutrition.

## 1. Introduction

Malnutrition is characterized by deficiencies, excesses, or imbalances in the intake of energy and/or nutrients. In this context, undernutrition manifests in four broad forms: wasting, stunting, underweight, and micronutrient deficiencies, affecting growth, development, and overall health of children around the world. It is estimated that in 2022, around 45 million children under the age of 5 (6.8%) were affected by wasting, with 13.6 million (2.1%) suffering from severe wasting. Over three-quarters of children with severe wasting live in Asia, while 22% are in Africa [1,2].

Childhood undernutrition is the result of a complex interaction of individual, socioeconomic, and environmental factors, which increases children’s vulnerability to diseases and mortality [2]. Among these conditions, diarrhea stands out as the second leading cause of death in children under 5, especially in economically disadvantaged populations, despite a one-third reduction in mortality over the past decade.

The relationship between undernutrition and intestinal infectious diseases, such as diarrhea, is mediated by a vicious cycle that exacerbates damage to child health. Intestinal infections, including both acute and chronic episodes, contribute to enteric dysfunction, characterized by intestinal inflammation, nutrient malabsorption, and altered intestinal permeability [3,4]. Additionally, the translocation of microbial components from the lumen or intestinal mucosa into the circulation and dysregulated immune responses further worsen the undernutrition condition [5,6,7]. These processes create an environment in which undernutrition weakens the immune system, increasing the risk of infections, while intestinal infections worsen undernutrition, perpetuating the cycle and compromising child growth and development [8].

The assessment of intestinal permeability has been useful in analyzing the integrity of the barrier function in children with undernutrition, especially in tropical regions of developing countries, where growth delays and altered anthropometric indicators (such as height-for-age, weight-for-age, and weight-for-height) have been associated with increased intestinal permeability. These changes reflect morpho-functional damage to the small intestine, with a negative impact on nutrient absorption and child growth [9,10,11].

In this review, we explore the main factors associated with childhood undernutrition, with a focus on intestinal infections and key biomarkers of altered intestinal permeability that impair child development. Although global mortality from diarrheal diseases has significantly declined since 1990, children under five years old still bear the greatest burden, accounting for 79% of the 1.2 million deaths attributed to these illnesses [12]. This enduring vulnerability highlights the continued relevance of enteric diseases as a public health concern and reinforces the need for renewed attention and innovative therapeutic strategies. Given the high prevalence of undernutrition and its strong association with intestinal infections—particularly in tropical and socioeconomically vulnerable regions—it is crucial to investigate the mechanisms that perpetuate this cycle. In this context, the assessment of intestinal permeability emerges as a valuable tool for understanding functional gut damage and its impact on child growth. By identifying early alterations in the gut associated with undernutrition, this study aims to support more effective health interventions, emphasizing the critical role of nutrition in breaking this cycle.

## 2. Methods

The narrative review was conducted using the scientific databases Medline/PubMed, Web of Science, and Scopus, from July 2024 to March 2025. The data search was performed using a combination of keywords related to childhood undernutrition in children under five years of age, intestinal infections, and markers of intestinal permeability. Titles and abstracts were initially screened to identify studies that met the inclusion criteria; those considered relevant proceeded to full-text reading for final selection. There were no language restrictions. Studies were excluded if they lacked therapeutic actions directly related to the research focus, or were categorized as books, book chapters, case reports, editorials, letters, dissertations, or theses. As this is a narrative review, registration on global platforms such as PROSPERO was not required.

## 3. Undernutrition in Childhood (Epidemiology, Etiology, General Mechanisms)

Malnutrition in childhood can be attributed to a complex interaction of individual, socioeconomic, and environmental factors, making children more vulnerable to illness and death [2]. Considering undernutrition, poverty is known to amplify its risk. In this sense, insufficient access to food, poor quality complementary feeding, inadequate feeding practices, and increased infection rates are potential risk factors for stunting [2,13,14]. Moreover, persistent enteric infections compromise absorption and intestinal barrier functions and result in up to 43% growth retardation [15].

Currently, approximately 45 million children in the world are wasting, and 39 million children are overweight. Stunting affects 148.1 million children under the age of 5 and has decreased significantly in the last decade; however, it is still present in 22.3% of children in this age group worldwide [1]. Undernutrition is linked to almost half of the deaths among children under 5 in underdeveloped countries [2] where the lack of drinking water and adequate sanitation allow high rates of enteric infections and diarrhea to persist [15]. Africa and Asia collectively share the most significant numbers of all forms of undernutrition [16]. In 2022, 70% of all children under five affected by wasting lived in Asia, and more than a quarter lived in Africa (WHO/UNICEF; 2023 [1]). According to the WHO (2009), wasting is defined when weight-for-height is <−2 *z*-scores, stunting when height-for-age is <−2 *z*-scores, and overweight when body mass index (BMI) is ≥3 *z*-scores [17].

Although the last decade has seen an improvement in maternal and child health and nutrition [2] undernutrition is still prevalent, and compromises physical, cognitive, and immunological functional capacity [3], as well as altered central nervous system (CNS) development and other abnormalities in children. Undernutrition is associated with an increased mortality risk due to infectious diseases, including pneumonia and diarrhea [18]. Diarrhea due to environmental enteric dysfunction in early childhood is associated with reduced height and weight in the first five (5) years of life [19].

Among the mechanisms of undernutrition, systemic and intestinal inflammation plays an important role [13]. Systemic inflammation involves the release of cytokines such as tumor necrosis factor-alpha (TNF-α), IL-1, IL-6, and IL-12 that can negatively influence body composition through reduced appetite, with direct catabolic effects on skeletal muscle and adipose tissue [20,21], as well as altered growth hormone and insulin-like growth factor 1 (IGF-1) resulting in impaired linear growth [5]. Increased activation of the ubiquitin–proteosome pathway is the primary process that degrades myofibrillar proteins in conditions of food shortage [20,22,23].

Concerning intestinal inflammation, immune activation in undernutrition can be caused by acute and chronic infection, intestinal enteropathy, translocation of microbial components from the intestinal lumen or mucosa into the circulation [5,6], and deregulated immune responses [7]. In this context, we can highlight the infection/undernutrition vicious cycle, which involves multiple pathways such as intestinal inflammation, malabsorption of nutrients, and altered intestinal permeability [3,4]. The integrity of the gastrointestinal barrier and mucosa is often impaired in children with undernutrition, and the effects of this impairment are aggravated by chronic subclinical enteric dysfunction in close association with alterations in the intestinal microbiota [24,25,26].

Diarrhea in undernourished children is triggered by intestinal infections and inflammation [27] and is associated with unfavorable clinical outcomes [28,29,30]. In addition, poor digestion of nutrients resulting from impaired hepatobiliary and pancreatic exocrine function can contribute to malabsorption of nutrients and aggravate diarrhea [31,32]. Undernutrition has repercussions on flattening the small intestine’s villi, reducing the absorption capacity mainly of monosaccharides and disaccharides, which can contribute to osmotic diarrhea [33].

Other alterations in undernutrition during childhood are altered macronutrient metabolism and endocrine function [34,35], modified glucose homeostasis [36], increased oxidative stress with reduced levels of antioxidants such as vitamin E and glutathione [37], altered cardiac function with atrophy of the heart muscle and reduced cardiac output [38], and liver dysfunction with non-alcoholic hepatic steatosis [39] as a result of mitochondrial dysfunction [40].

**Figure 1:**
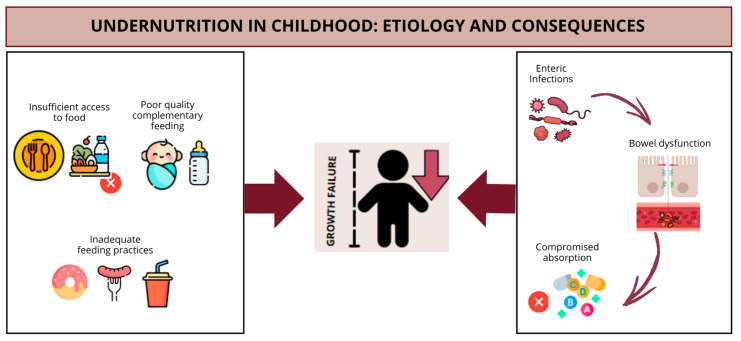
Etiological factors and consequences of undernutrition in children.

Child undernutrition results from a complex interaction between individual and socio-environmental factors and is aggravated by poverty, which compromises children’s growth and development. Undernutrition is associated with almost half of deaths in children under five years of age, mainly in underdeveloped countries, where a lack of basic sanitation perpetuates enteric infections. Systemic and intestinal inflammatory mechanisms contribute to nutrient malabsorption, loss of muscle mass, and hormonal changes that affect growth. The etiology and consequences of undernutrition are summarized in Figure 1. 

## 4. Intestinal Infections in Children

Diarrhea is the third cause of mortality in children from 1 to 59 months of age, killing about 443,832 children under 5 years. Untreated water, lack of basic sanitation, and inadequate personal hygiene are the main causes of children’s diarrhea [2]. Furthermore, exclusive breastfeeding was associated with a lower likelihood of detecting four important enteric pathogens in the first 6 months of life and delays in the initial detection of some bacterial and viral pathogens in stool [41]. Climatic conditions are also reported to influence gastroenteritis. Temperature, soil moisture, and humidity influence infections by several enteropathogens, probably impacting the survival of the pathogen outside the host [42].

Children with undernutrition or immunological problems too have a greater risk of death. Sub-Saharan and southern Asia and Africa are the regions with the highest child mortality rates due to diarrhea [1]. In addition, low-income countries have higher diarrhea incidences, with more than three episodes per year in children under 3 years of age, which increases the risk of undernutrition.

Diarrhea is characterized by the occurrence of three or more loose or watery stools in a single day, or an increased frequency compared to an individual’s normal pattern. There are three clinical types of diarrheas: (1) acute aqueous diarrhea, which can last several hours or days and includes cholera; (2) acute and bloody diarrhea, which is also called dysentery; and (3) persistent diarrhea, which lasts 14 days or more [2].

Enteric childhood infections, especially diarrheal, are worrying not only for their association with child mortality, but also for generating malabsorption and undernutrition, especially when persistent. Undernourished children have a compromised immune response, increasing their susceptibility to enteric infections, generating a vicious cycle of enteric infections and undernutrition [43]. This vicious cycle has long-term consequences such as cognitive deficit and metabolic syndromes in adulthood. Thus, more appropriate treatments and prevention methods should be applied to avoid long-term impact.

Several microorganisms such as viruses, bacteria, or protozoa may cause diarrhea [2]. Identifying the etiology of enteric infections is a great challenge, especially for the variety of pathogens involved and the sensitivity of diagnostic techniques. The reanalysis of the Malnutrition and the Consequences for Child Health and Development (MAL-ED) cohort study’s enteropathogen diagnosis using the quantitative molecular diagnostic method improved estimates of the specific pathogen load in childhood diarrhea in community settings [44] compared to the previous study that used conventional microbiology diagnosis [45]. Next, we will detail the prevalence and outcome of childhood gastroenteritis caused by viruses, bacteria, and protozoans.

### 4.1. Virus

Viruses are generally considered the main cause of childhood diarrhea in high-income countries [46]. However, the MAL-ED study, which evaluated diarrheal stools from children in eight low-income countries, showed that viral diarrhea was more common (36%) than bacterial diarrhea (25%) and parasitic diarrhea (3.5%) in the first two years of life [47]. The most common viruses in diarrheal samples in this study were the following, in order of highest to lowest incidence: rotavirus, adenovirus 40/41, sapovirus, norovirus, and astrovirus (0–11 months of age); and sapovirus, rotavirus, adenovirus 40/41, astrovirus, and norovirus (12–24 months of age) [47]. In the first year of life, rotavirus was the first most prevalent pathogen, while in the second year of life rotavirus was the fourth and sapovirus became the second most prevalent pathogen [47].

In contrast, the most prevalent virus in 1729 diarrhea samples from children up to 2 years of age in a placebo-controlled clinical trial of the Rotasiil vaccine in Niger was rotavirus [47], while a study of 2692 children in Shanghai found that acute diarrhea was mainly caused by rotavirus and norovirus [48]. In the latter study, the most common viruses were rotavirus (16.0%), followed by norovirus (15.5%), adenovirus (7.2%), sapovirus (3.0%), and astrovirus (2.7%) [48]. In a cross-sectional, case–control, age-matched study of children aged 2 to 36 months from six cities in the Brazilian semiarid region showed that norovirus GII, adenovirus, and rotavirus were the predictive viruses for diarrhea [49].

Besides rotavirus, other viruses have gained importance in the etiology of pediatric diarrhea. Among them, rotavirus remains one of the main causes of acute gastroenteritis worldwide, being associated with high rates of hospitalization and infant mortality. In low-income countries, rotavirus diarrhea is frequently linked to fever, vomiting, dehydration, and higher severity [44]. Among the circulating genotypes, G1P [8] is the dominant strain globally [50,51]. Although vaccination significantly reduces disease burden [44,50,52,53,54], vaccine failure in low- and middle-income countries remains a concern [55]. Environmental factors such as runoff, early complementary feeding, nutritional status, dehydration, and age under two years have also been associated with increased risk [42,56,57].

Sapovirus has gained relevance in post-rotavirus vaccination settings. As previously mentioned, it was the most prevalent virus in diarrheal samples during the second year of life in low-income countries [44]. It has been reported in 1 to 17% of diarrhea episodes globally, especially in young children and older adults [58]. In hospitalized children under five years of age, sapovirus infection accounted for 8% of cases [59]. An epidemiological study revealed that over 90% of children in eight low-income countries were infected with sapovirus, and 60% experienced sapovirus-associated diarrhea within the first two years of life [60]. Protective factors included breastfeeding and higher socioeconomic status. Prior infection reduced the risk of reinfection by 22% and subsequent diarrhea by 24% [60].

Unlike sapovirus, norovirus GII has been associated with growth faltering. A study conducted in the Brazilian semiarid region detected norovirus in 45.2% of diarrheal samples from children aged 2 to 36 months [61]. Norovirus infection was linked to lower z-scores for weight-for-age, weight-for-height, and BMI-for-age. Additionally, respiratory symptoms were more frequent in norovirus-positive individuals, and a high prevalence (80%) of recombinant strains was observed [61].

Adenovirus 40/41 also plays a significant role in children under six months of age in low-income settings [62]. It was more likely to cause fever than norovirus, sapovirus, and astrovirus, though not more than rotavirus. Norovirus and rotavirus were more commonly associated with vomiting [44]. Exclusive breastfeeding was the only identified protective factor [62]. Astrovirus, in turn, infected 35% of children in the MAL-ED study across eight countries [63]. Its severity surpassed that of most other enteropathogens, second only to rotavirus. Malnutrition, particularly stunting, was a key risk factor for astrovirus diarrhea [63].

Interestingly, one study found that viral diarrhea episodes were more frequently associated with a positive trend in cognitive scores among 6- to 8-year-old children from three MAL-ED sites, although not statistically significant [64]. However, adenovirus 40/41 infection was linked to lower performance in semantic and phonemic fluency tasks, while norovirus GII and sapovirus were associated with poorer reasoning skills [64].

It is important to note that post-COVID-19 pandemic studies have reported an increase in acute gastroenteritis caused by other viruses. For example, enterovirus was detected in 8.8% of fecal samples from children hospitalized in Thailand [65]. Another study demonstrated a high prevalence of norovirus GII, adenovirus, and rotavirus A, followed by human parechovirus, enterovirus, and sapovirus (SaV) in 152 fecal samples collected from children hospitalized for gastroenteritis in Italy [66].

Therefore, enteric viruses continue to be important causes of childhood diarrhea worldwide, especially rotavirus, sapovirus, norovirus, adenovirus 40/41, and astrovirus. The distribution of these agents varies according to age group, geographic region, and vaccination coverage. Viral infections influence not only morbidity but also the physical and cognitive development of affected children. Although vaccination has reduced rotavirus morbidity, the emergence of other viruses highlights the importance of integrated strategies for prevention, surveillance, and management of intestinal viruses in childhood, especially in contexts of social vulnerability.

### 4.2. Bacteria

Bacteria responsible for enteric infections in children can lead to diverse outcomes, including both clinical and subclinical manifestations, associations with growth faltering, and even increased risk of mortality. Studies have shown significant heterogeneity in bacterial prevalence depending on geographic region, diagnostic methods, and diarrhea severity.

For instance, the Global Enteric Multicenter Study (GEMS), conducted among 9439 children under five years of age with moderate-to-severe diarrhea across four African and three Asian countries, identified *Shigella* and heat-stable enterotoxin-producing *Escherichia coli* (ST-ETEC) as the most prevalent bacterial pathogens [67]. Other bacteria, such as *Aeromonas*, *Vibrio cholerae* O1, and *Campylobacter jejuni*, showed regional significance [67]. Similar results were found in a study that evaluated diarrhea samples from children up to 2 years of age, belonging to the placebo study of the Rotasiil vaccine in Niger [47]. Notably, ST-ETEC and typical enteropathogenic *E. coli* (EPEC) carrying the eae and bfp genes were significantly associated with infant mortality in the first year of life [67].

In a separate study conducted in Shanghai (2015–2018) using culture-based diagnostics, nontyphoidal *Salmonella* (NTS) was the most frequently isolated bacterium (10.3%), followed by EPEC (6.5%), enteroaggregative *E. coli* (EAEC, 6.2%), *Campylobacter* (3.6%), ETEC (1.1%), *Shigella* (0.2%), and enterohemorrhagic *E. coli* (EHEC, 0.1%) [48]. However, the primarily culture-based diagnostic approach may underestimate the presence of fastidious bacteria.

Data from the MAL-ED cohort, which analyzed 6625 diarrheal stools from children aged 0–24 months, highlighted ETEC, *Campylobacter jejuni*/*coli*, *Shigella*, typical EPEC, EAEC, and atypical EPEC as leading bacterial pathogens in infancy, with slight shifts in prevalence during the second year of life [44]. Pathogen prevalence also varied with geography, season, and diarrhea severity. *Campylobacter* and *Shigella* were particularly associated with bloody diarrhea, with *Shigella* becoming the leading pathogen by the second year of life [44].

Among these, *Shigella* has received growing attention due to its high detection rates in both symptomatic and asymptomatic children. In the MAL-ED study, 75.5% of children had at least one non-diarrheal sample positive for *Shigella*, and 29.6% experienced at least one diarrheal episode attributed to it [68]. In Niger, *Shigella* accounted for over 60% of severe shigellosis episodes in infants aged 12–23 months [47]. The shedding of *Shigella* post-diarrhea is prolonged [69], increasing its transmission potential. The most frequently identified serotypes were *S. sonnei* (30.4%), *S. flexneri* 2a (20.8%), and S. flexneri 6 (24.6%), the latter associated with milder infections. Importantly, asymptomatic *S. flexneri* was also linked to impaired linear growth [70].

Additionally, *Shigella* infection was associated with long-term developmental effects, including lower height-for-age *z*-scores at 6 and 8 years, and decreased verbal fluency, particularly in Brazilian children [68]. High *Shigella* burden was also associated with reduced semantic fluency across sites in Brazil, Tanzania, and South Africa, suggesting region-specific cognitive effects [64]. Environmental risk factors such as high rainfall, soil moisture, elevated temperatures, and inadequate sanitation increase susceptibility to *Shigella* infection [71].

*Campylobacter* is another highly prevalent enteropathogen. In MAL-ED, it was detected in 84.9% of 1892 children within their first year of life [72]. Persistent infections, defined as three or more positive stools per month, occurred in nearly half the cohort and lasted on average 150 days [73]. These persistent infections were associated with impaired growth at 24 months and higher levels of intestinal and systemic inflammation (e.g., elevated fecal MPO, A1AT, and serum AGP) [72]. Furthermore, subclinical *Campylobacter jejuni* infection showed a specific profile of virulence genes, and increased systemic response in malnourished children [74]. *Campylobacter* has also been linked to cognitive deficits, similar to *Shigella* and some viral infections [64].

ETEC and EPEC are also prominent contributors to both the severity and long-term consequences of enteric infections in young children. In a longitudinal birth cohort study conducted in Peru, ETEC was identified in multiple diarrheal episodes in nearly 70% of children, with infections occurring as early as the neonatal period and increasing in prevalence during the second year of life [75]. Among these, ST-ETEC was particularly associated with recurrent infections, and episodes lasting five days were significantly linked to stunting and wasting within the following 30 days. Similar findings were observed in Bangladesh, where children who were stunted or at risk of stunting had a high prevalence of fecal ETEC, correlating with increased markers of intestinal inflammation [76]. EPEC also demonstrated a strong association with undernutrition in this population. Supporting evidence from other studies indicated that atypical EPEC was significantly associated with higher environmental enteropathy scores—a condition that contributes to growth faltering—while typical EPEC was directly linked to impaired linear growth at 24 months of age [77]. Furthermore, EPEC has been found in both diarrheal and asymptomatic samples, underscoring its widespread circulation and potential for subclinical impact in early childhood [78].

Although initially considered more prevalent, EAEC’s importance remained even after molecular diagnostics revised its estimated frequency downward. While conventional microbiology ranked it among the top three bacterial pathogens, quantitative molecular diagnostics placed EAEC 11th [44]. Nevertheless, EAEC was detected in 27.5% of diarrheal and non-diarrheal samples, with 94.8% of children testing positive by two years of age [79]. EAEC was associated with subclinical intestinal inflammation, and reduced length-for-age *z*-scores. Moreover, early-life co-infections involving EAEC were associated with dysregulated immune responses and increased risk of undernutrition [80].

In summary, bacterial enteric infections—particularly those caused by *Shigella*, Campylobacter, ETEC, EPEC, and EAEC—pose a substantial burden in early childhood. These pathogens contribute not only to acute illness but also to long-term consequences such as stunting, chronic inflammation, and cognitive impairment. The high rates of asymptomatic colonization, geographic variability, and complexity of co-infections underscore the need for integrated approaches combining surveillance, accurate diagnostics, and context-specific prevention strategies. Notably, the overlooked clinical significance of EAEC, due to its frequent subclinical presentation, highlights the importance of addressing pathogens beyond those typically associated with overt diarrhea.

### 4.3. Protozoan

Protozoan are present in less than 5% of cases, especially *Cryptosporidium* spp., *Giardia lamblia,* and *Entamoeba histolytica*, being more frequent in medium- and low-income countries, with poor sanitization and hygiene, as well as bacteria [81].

*Entamoeba histolytica* is a protozoan parasite and the causative agent of amebic dysentery. The infection is transmitted through the fecal–oral route, typically via contaminated water or food, and is particularly prevalent in sub-Saharan Africa and South Asia. It is characterized by diarrhea and abdominal pain and can lead to more severe complications such as liver abscesses. In children, the infection can range from asymptomatic colonization to moderate-to-severe diarrhea, leading to significant morbidity [82,83].

The relationship between intestinal infection in children and *E. histolytica* is influenced by several factors. Colonization by *E. histolytica* has been inhibited by the presence of mucosal IgA antibodies against the pathogen’s adherence lectin, highlighting the role of the immune response in combating infection and suggesting a basis for vaccine development [84,85]. Genetic factors may also play a role in the severity of *E. histolytica* infections through specific polymorphisms and loci, particularly involving the CREM and CUL2 genes. Lower expression of these genes has been linked to greater susceptibility, while their upregulation may offer protection against severe outcomes in the early stages of the disease [86].

The severity and progression of *E. histolytica* infection are also influenced by the gut microbiota. In a study conducted by Gilchrist, C. A. et al. (2016) [85] with 392 children born into an urban slum in Dhaka, 80% of the children in the Mirpur slum had *E. histolytica* infections, with 17% of these resulting in diarrhea. Fecal IgA antibodies against the anti-galactose/N-acetylgalactosamine lectin were associated with protection from reinfection, while *Prevotella copri* levels were associated with diarrhea.

In a case–control study conducted across six cities in Brazil, involving 1200 children aged 2–36 months, viral pathogens were detected in 186 cases, bacterial pathogens in 1107 cases, and protozoan pathogens in 188 cases among children with diarrhea. These frequencies represent the total number of positive detections, which may include co-infections; as such, individual children may have been counted more than once within each group [49].

*Giardia* lamblia is another protozoan that commonly infects children, causing giardiasis. This infection can be asymptomatic or lead to diarrhea and malabsorption, which may negatively affect growth and development [87]. Malabsorptive diarrhea caused by *Giardia* is a significant contributor to stunting in young children, as it can alter intestinal villi, epithelial cells, the mucous barrier, and intestinal permeability. These alterations have been associated with impaired growth, reflected by reduced height-for-age (HAZ) and weight-for-age (WAZ) *z*-scores. Moreover, Giardia infections are often recurrent, contributing to a cycle of persistent diarrhea and malnutrition [88].

Paradoxically, *Giardia* has also been observed to exert a protective effect against moderate-to-severe diarrhea (MSD). This suggests that Giardia may influence the colonization or infection by other enteric pathogens, thereby affecting the clinical outcomes of intestinal infections. Specifically, *Giardia*-induced changes in the gut microbiome may create an environment less conducive to the invasion or colonization by other pathogens. This potential protective effect seems to be particularly relevant in low-resource settings, where multiple enteric infections are [89,90]. In contrast, *Giardia* has also been associated with post-infectious complications, including irritable bowel syndrome (IBS), chronic fatigue, and lactose intolerance [90,91].

*Giardia* was associated with persistent infection in the first 6 months of life, being related to increased intestinal permeability and failure to thrive at 2 years of age in the MAL-ED study [49]. Similar results regarding *Giardia* infection were found in a longitudinal prospective cohort study carried out in Bangladesh [92].

*Cryptosporidium* spp. are also important pathogens, especially in immunocompromised individuals. Like other protozoan infections, cryptosporidiosis can range from asymptomatic to causing severe diarrhea, leading to dehydration and malnutrition, particularly in children under five years of age. It has been associated with negative impacts on growth indicators, including decreases in height-for-age *z*-scores (HAZ; stunting), weight-for-age *z*-scores (WAZ; underweight), and weight-for-height z-scores (WHZ; wasting) [93].

The prevalence of *Cryptosporidium* infection varies depending on geographic location and population characteristics. In the United States, serological surveys have shown a prevalence of 21.3% among children under 10 years of age [94]. In low-income settings across Africa, Asia, and South America, the MAL-ED study reported that 65% of children were affected [95]. In China, a prevalence of 2.9% was observed among children, with higher rates found in the southwestern regions and among those under three years old [96].

These findings highlight the complexity of the host–protozoan interaction and underscore the need for comprehensive public health strategies to mitigate the impact of this infection, especially among vulnerable populations.

### 4.4. Co-Infections

Co-infection refers to the simultaneous infection of a host by multiple pathogenic organisms, which can alter disease outcomes through synergistic or antagonistic interactions. In recent years, co-infections have gained increasing attention due to their frequent occurrence in both clinical and subclinical enteric infections, particularly in low- and middle-income settings. A retrospective study analyzing diarrheal samples collected between 2014 and 2016 from hospitals and primary care facilities in sub-Saharan Africa found that 59% of samples involved co-infection [97]. Among these, 53% harbored two pathogens, 22% three pathogens, and 25% four or more [97]. Most studies have not adequately addressed the impact of co-infections on disease severity and persistence, although they are often linked to prolonged diarrheal episodes.

Several studies have investigated specific pathogen combinations in co-infections. For example, sapovirus-positive samples from children were frequently coinfected with rotavirus, astrovirus, adenovirus, and *Shigella*, in both diarrheal and non-diarrheal stools [60]. Rotavirus co-infections are also commonly observed across different regions. In the Brazilian semiarid region, EAEC was the most prevalent co-pathogen with rotavirus [50], whereas in Bangladesh, co-infections involving ETEC and rotavirus were particularly frequent in children under five [98]. In the same Brazilian study, EAEC and norovirus co-infection was a significant predictor of diarrhea, while *Shigella* was frequently associated with norovirus-positive cases [61].

Beyond their role in acute disease, co-infections have also been associated with poor nutritional outcomes and intestinal inflammation. Data from the MAL-ED cohort study demonstrated that co-infections in non-diarrheal samples from infants aged 0–6 months were negatively associated with weight-for-length and weight-for-age Z-scores, and positively associated with elevated fecal myeloperoxidase (MPO) levels, an inflammatory marker [80]. EAEC was the most frequently identified pathogen in these co-infections, often appearing alongside *Campylobacter*, heat-labile toxin-producing ETEC, *Cryptosporidium*, and atypical EPEC [80].

In summary, co-infections are highly prevalent in pediatric enteric disease and may play a crucial role in shaping disease severity, persistence, and nutritional outcomes. Despite this, the clinical relevance of co-infections remains underexplored, especially in terms of their contribution to inflammation and growth impairment. The frequent involvement of EAEC, EPEC, and ETEC in co-infection scenarios—even in the absence of diarrhea—emphasizes the need for improved diagnostics and comprehensive longitudinal studies. Future research should aim to clarify the complex interactions among co-pathogens and how they influence child health, particularly in settings where enteric disease, undernutrition, and environmental enteropathy intersect. Table 1 summarizes the main findings of enteric infections in children and their clinical outcomes.

## 5. Undernutrition and Environmental Enteropathy: From Epidemiological Studies to Experimental Models

**Figure 2:**
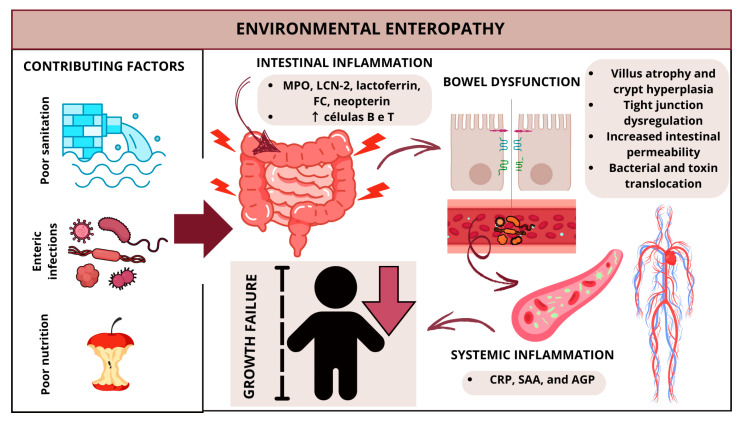
Contributing factors and physiological changes during environmental enteropathy. Poor sanitation, enteric infections, and poor nutrition are environmental factors that contribute to development of EE. These environmental factors cause recurrent insults to the small intestine, which generate intestinal inflammation (increased MPO, LCN-2, lactoferrin, FC and neopterin, increased B and T lymphocytes) and intestinal dysfunction, such as villous atrophy and crypt hyperplasia, as well as increased intestinal permeability that generates bacterial and toxin translocation. This translocation favors development of systemic inflammation that ultimately culminates in growth deficit.

Environmental enteric dysfunction (EED), also known as environmental enteropathy (EE), is a chronic, subclinical intestinal condition strongly associated with childhood growth restriction [100]. In general, EE is defined by small intestine injury, impaired nutrient absorption, and microbial translocation, which together trigger chronic systemic inflammation and ultimately contribute to stunting [100] (Figure 2).

The etiology of EE remains poorly understood. However, evidence from large-scale longitudinal studies, such as the MAL-ED cohort, as well as experimental models, suggests a multifactorial origin involving recurrent enteric infections, inadequate complementary diets, and compromised immune regulation. For instance, a low fiber intake in complementary diets has been associated with higher EE scores in children aged 9 to 15 months in the MAL-ED cohort study [84]. Enteric infections caused by norovirus, *Campylobacter*, LT-ETEC, *Shigella*, *Giardia*, EAEC, EPEC, ETEC, *Shigella*/enteroinvasive *E. coli*, Shigatoxigenic *E. coli* and *Cryptosporidium*, in isolation, are more strongly associated with growth restriction [48,53,74,76]. Notably, non-diarrheal [24] and subclinical infections, particularly involving EAEC, also contribute to linear growth impairment in EE [80].

Alterations in the small intestine microbiota have also been associated with EE and stunting, although it remains unclear whether this is a cause or consequence of EE. A study found that high bacterial loads in the upper gastrointestinal tract (>10^5^ CFU/mL) were linked to poor growth at two years of age [91]. Moreover, gene expression analyses of duodenal biopsies from children with EE revealed upregulation of immune-related pathways such as IL-17 and JAK-STAT signaling, and downregulation of antioxidant and detoxification pathways [101].

EE is generally characterized by specific alterations in the upper small intestine, such as villous atrophy with frequent crypt hyperplasia [102]; increased intestinal permeability [103,104,105] tight junction alterations [106]; increased infiltration of activated B and T cells [102]; immune dysregulation [107,108]; and elevated interferon-gamma (IFN-γ) with reduced interleukin-10 (IL-10) levels [109] in the lamina propria. Biopsies from Pakistani children with EE showed partial villous atrophy, a marked increase in intraepithelial lymphocytes, increased claudin-4, and upregulated expression of nutrient, water, and basolateral chloride (NKCC1) transporters [106], suggesting an adaptation of the intestinal epithelium to insufficient nutrient absorption for restoring affected individuals’ health.

Because EE is asymptomatic, efforts have focused on developing surrogate biomarkers. A histopathological scoring system with high discriminatory power for EE was developed in duodenal biopsies from DEE cohorts in Bangladesh, Pakistan, and Zambia [110]. This scoring system is based on blunted villous architecture, increased intraepithelial lymphocytosis, goblet cell depletion, Paneth cell depletion, and reduced intramucosal Brunner’s glands.

Increased biomarkers of impaired barrier function and intestinal epithelial damage, such as elevated lactulose:mannitol (L:M) ratio, fecal α1-antitrypsin (A1AT), fecal Reg-1, and plasma zonulin; bacterial translocation markers, such as anti-LPS antibodies or circulating LPS; intestinal inflammation markers, such as MPO, LCN-2, lactoferrin, FC, and neopterin; systemic inflammation markers, such as CRP, SAA, and AGP; and endocrine/metabolic markers, such as growth hormone and insulin-like growth factor-1 (IGF-1), have all been associated with EE [100] (Figure 2). However, these biomarkers do not always correlate, and different combinations may predict distinct outcomes, such as systemic inflammation or poor absorptive capacity [5,111,112].

In vivo experimental models serve as crucial tools for better understanding the complexity of this pathology. Protein-deficient diets have proven highly relevant for EE establishment, as they impair mucosal homeostasis. However, protein deficiency alone is insufficient to explain all EE characteristics.

Studies using moderately or severely protein-deficient diets demonstrate variable alterations in tight junction proteins with consistent changes in intestinal permeability [113,114,115]. Villous lengths are reduced with increasing protein deficiency [113,116], but crypt hyperplasia is not always observed. Intestinal epithelial cell proliferation and apoptosis are also impaired by protein malnutrition, explaining the reduced host defense [117]. Undernutrition also decreases mucus layer depth, goblet cell number, mucus content within goblet cells, and goblet cell differentiation [118], corroborating reduced mucin gene expression in children with EE [119], which may be related to bacterial translocation [112].

Innate and adaptive mucosal immune responses are also altered by nutritional deficiencies. For example, protein malnutrition increases inflammatory markers (MPO and LCN-2) [120], toll-like receptor (TLR)2 and TLR4 expression [121], and intraepithelial lymphocytes [96], while decreasing T and B cells in the lamina propria [122] and serum levels of acute-phase reactant serum amyloid A [100]. CD4+ T cells, however, are disproportionately reduced in both protein malnutrition and zinc deficiency [100]. Nevertheless, the reduction of lamina propria lymphocytes during malnutrition contrasts with EE characteristics, suggesting that additional factors are necessary for EE establishment.

Zinc deficiency is also associated with EE [123] and stunting [124] in children, although in experimental models it is not sufficient to induce EE independently. Zinc deficiency alone does not impair growth in mice [120], does not cause intestinal architectural alterations or barrier disruption, but limits Paneth cell function [125] and exacerbates weight loss, fecal pathogen load, and intestinal damage markers during EAEC [7,126], ETEC [8], *Shigella* [127], and *Campylobacter* infections [19].

A nutrient-, energy-, and zinc-deficient diet increased crypt depth in all intestinal segments over a chronic period, associated with increased paracellular permeability and growth restriction in recently weaned mice [31]. However, this model showed villous blunting only in the mid-intestine, without increased L/M ratio, impaired nutrient absorption, or visible histopathological changes over the chronic period [31], further confirming that diet alone is insufficient to fully reproduce the characteristic EE phenotype in experimental models.

Experimental models of nutritional deficiency and infection with specific pathogens induce some, but also not all, EE characteristics. Administration of an *E. coli* and Bacteroidiales cocktail [113], Giardia [128], EAEC [129], or *Cryptosporidium* [130] can exacerbate villous blunting during protein malnutrition; however, significant crypt hyperplasia is observed only with *Cryptosporidium* infection [116]. Intestinal permeability is impaired only in infections with *E. coli*/Bacteroidiales mix [113], or *Giardia* [131]. *C. parvum* oocysts inoculated during protein malnutrition increased CCL5 chemokine levels, IFNg, and B and T cell recruitment to the lamina propria [113]. Protein or zinc deficiency combined with EAEC 042, ETEC, and *Campylobacter* challenge results in heightened myeloid cell responses [122,132,133,134], consistent with increased fecal MPO in malnourished children exposed to bacterial enteropathogens [135]. Only zinc-deficient models with *Campylobacter jejuni* and ETEC infections result in consistent diarrhea [133,134].

Many of these specific pathogen infection models require intestinal microbiota depletion and nutritional deficiency to induce EE-like characteristics in animals. Nutritionally fed gnotobiotic piglets inoculated with fecal microbiota from children with EE did not develop EE histopathological features but exhibited more severe rotavirus-induced diarrhea [136]. This confirms the necessity of an interaction between nutritional deficiency, dysbiotic microbiota, and intestinal pathogens for EE establishment, as observed in clinical studies. Additionally, studies have also reported a set of environmental toxins, called exposomes, as contributors to EE establishment [137,138,139], which could further explain why experimental models fail to fully reproduce EE characteristics.

Altogether, these findings reinforce that environmental enteropathy results from a complex and dynamic interplay of factors, requiring the interaction between malnutrition, enteric infections, intestinal microbiota, and possibly environmental toxins. Although there has been substantial progress in identifying biomarkers and generating animal models, current systems cannot fully replicate the human condition. Future research should integrate clinical, molecular, and environmental data to elucidate the mechanisms underlying EE and develop effective prevention and treatment strategies. Table 2 summarizes the main findings of the EE.

## 6. Intestinal Barrier Function and Undernutrition in Children

The failure to thrive among children under 5 years old is commonly reported as an issue, especially in tropical regions of developing countries. Linear growth deficit and anthropometric indicators such as height-for-age, weight-for-age, and weight-for-height have been associated with increased intestinal permeability in children residing in these areas. These changes are caused by morpho-functional alterations at the level of the small intestine, commonly associated with undernutrition in children [9]. Lee et al. (2017) [141], examined a large cohort of children and found that factors such as severe diarrhea and enteropathogenic exposure were associated with increased of intestinal permeability, using the lactulose:mannitol test.

The association between intestinal barrier function and undernutrition has been widely discussed, as damage to intestinal health can potentially contribute to poor nutrient absorption, leading to inadequate growth [9]. Therefore, investigating biomarkers with good specificity, capable of ensuring a more precise diagnosis for alterations in intestinal barrier function, is very important and is still being studied [10].

In this context, various techniques for analyzing intestinal permeability as an indicator of barrier function have been investigated. The main approaches include the assessment of urinary excretion of probe molecules, which allows examination of the size and concentration of these molecules as they cross the intestinal barrier, such as in sugar tests, including lactulose:mannitol. Additionally, another approach involves evaluating circulating levels of mucosal damage markers, such as zonulin and lipopolysaccharide (LPS). Other strategies involve in vitro measurement using cell lines or human biopsies, analyzing the expression of various tight junction proteins such as claudins, occludins, and zonula occludens. Finally, endoscopic measurements also constitute an important tool in this context [11].

Among the methodologies mentioned, the most widely used for measuring changes in intestinal permeability in children has been the lactulose:mannitol test, which is considered a useful, simple, non-invasive, and reliable test for estimating intestinal permeability [10].

Lactulose is a large molecule that is minimally absorbed by the intact small intestine. However, if there is a change in intestinal permeability, this disaccharide can cross the intercellular spaces and be eliminated by glomerular filtration without renal tubular reabsorption, allowing easy measurement in urine. On the other hand, mannitol is absorbed through transcellular pathways according to the absorption capacity of the small intestine, which is determined by the surface area. The reduction of microvilli decreases the uptake and subsequent urinary excretion of mannitol, which like lactulose, is filtered and not reabsorbed. Thus, the urinary excretion of disaccharides and monosaccharides and the ratio of their excretion form the basis for measuring intestinal permeability. After measuring the concentration of the probes in the urine, the results are expressed as the proportion of the percentage excretion of the ingested dose of lactulose and mannitol in urine (lactulose:mannitol ratio = % lactulose/% mannitol) [11,142].

Despite the widespread use of this marker, there are still limitations to its interpretation. These difficulties include aspects related to test preparation, such as the requirement for fasting, complete urine collection over an extended period, and maintaining adequate hydration levels, which can be potentially challenging for children under 5 years old. Additionally, there is a lack of methodological standardization among studies, difficulty in interpreting results, especially in the presence of concurrent diseases, and concerns about bacterial overgrowth in the small intestine and/or dysbiosis [142,143].

Furthermore, although averages of ≤0.12 [10], ≤0.07 [144,145], and ≤0.09 [146,147] for the lactulose:mannitol test, and a lactulose excretion percentage of <0.20% [103,147] have been used in some studies as reference values for normality, there is a lack of standardized reference values considering different populations and age groups [11]. In general, studies involving the pediatric population, which investigate intestinal permeability using the lactulose:mannitol test, use either 5 g of lactulose and 1 g of mannitol dissolved in 20 mL of water, or they perform adjusted calculations based on 250 mg/mL lactulose and 50 mg/mL mannitol, at a dose of 2 mL/kg, or 400 mg/mL lactulose and 100 mg/mL mannitol, at a dose of 2 mL/kg.

**Figure 3:**
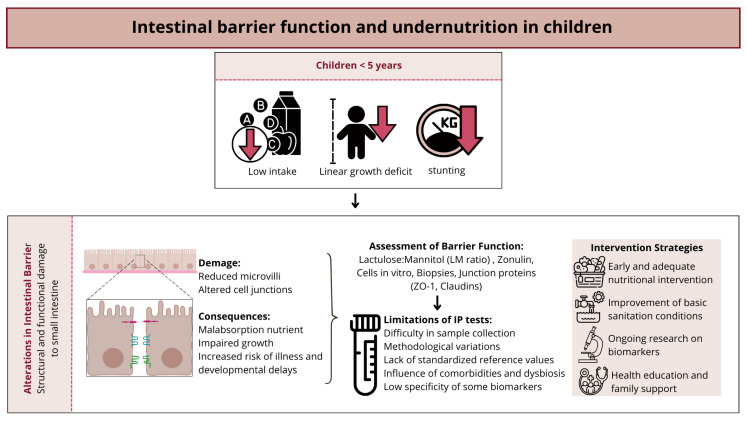
Intestinal barrier dysfunction and undernutrition in children: mechanisms, assessment, and intervention strategies.

In children, alterations in intestinal barrier function, particularly in those from underprivileged regions, are strongly associated with undernutrition and increased intestinal permeability. These changes, often linked to structural and functional damage to the small intestine, can lead to impaired nutrient absorption and subsequent growth deficits. Given the critical role of the intestinal barrier in maintaining overall health and development, it is essential to implement strategies that help restore and preserve its integrity. Early nutritional interventions, improved sanitation, and continuous research on reliable biomarkers for assessing intestinal permeability are crucial steps in addressing this issue. By ensuring a well-functioning intestinal barrier, it is possible to promote better nutrient absorption, support optimal growth, and reduce the risk of long-term health complications in children. The main aspects of intestinal barrier dysfunction in undernourished children—including pathophysiology, evaluation, and intervention—are visually summarized in Figure 3.

## 7. Nutrients and Intestinal Barrier Function in Children

Given the critical role of the intestinal barrier in nutrient absorption and overall health, targeted nutritional interventions are essential for its maintenance and restoration. Improving intestinal barrier function through strategic dietary modifications and nutrient supplementation can be a powerful approach to combating undernutrition and supporting healthy growth in children. A diet rich in cereals, beans, bananas, and pectin provides essential fibers and bioactive compounds that promote gut integrity, while key nutrients such as glutamine, vitamin A, zinc, and omega-3 PUFAs play a fundamental role in enhancing mucosal repair, modulating inflammation, and strengthening tight junctions. By integrating these nutritional strategies, it is possible to mitigate the adverse effects of increased intestinal permeability and foster long-term health improvements in children at risk [140,141,145,146,147,148,149,150,151,152,153,154,155,156,157,158,159,160,161,162,163,164].

Vitamin A, a fat-soluble molecule, belongs to the group of retinoids, which have biological activity. Recognized as a fundamental nutrient in maintaining intestinal health, vitamin A plays a crucial role in the regulation and differentiation of components of the immune system, both innate and adaptive, essential for the balance between immunogenicity and tolerance at the intestinal barrier [140,158]. Studies indicate that retinoids are involved in the regulation of gene expression related to intestinal epithelial barrier proteins, contributing to mucosal function and protection. Additionally, vitamin A plays a role in defense against enteric pathogens associated with intestinal diseases, especially in children with low intestinal permeability [159]. Morphological analyses in animal models have demonstrated that reduced levels of vitamin A are associated with decreased villus height, crypt depth, and reduced gene expression of tight junction markers. Consequently, low levels of vitamin A compromise intestinal epithelial integrity, increasing permeability and predisposing to inflammation [160].

In vitro and in vivo studies have been conducted to investigate the association between zinc and intestinal barrier function. Zinc deficiency leads to upregulated expression of intestinal uroguanylin, a peptide that stimulates chloride secretion and consequent intestinal water secretion, resulting in increased intestinal motility and diarrhea. Additionally, low levels of this nutrient decrease the absorption of triglycerides by impairing chylomicron formation and decrease the activity of enterocyte peptidase, leading to impaired protein absorption, amino acid absorption problems, and possibly damage to the intestinal mucosa. This deficiency can result in decreased expression of tight junction proteins, leading to increased intestinal permeability, which may allow harmful substances to pass through the intestinal barrier. Similarly, excessive serum zinc has been associated with pro-inflammatory effects in the intestine, which can further exacerbate intestinal conditions. Imbalances in zinc levels, whether due to deficiency or excess, impair the intestinal barrier and increase susceptibility to diseases [161,162].

Glutamine is an important energy source for the proliferation and differentiation of intestinal epithelial cells and appears to have a specific impact on the regulation of tight junction proteins, such as claudin-1. In addition to influencing intestinal permeability, glutamine can also affect nutrient absorption by the intestine and plays a crucial role in regulating various cellular pathways, including the inflammatory response, oxidative stress, and innate immune response, which collectively contribute to the modulation of permeability [163,164].

Omega-3, especially EPA (eicosatetraenoic acid) and DHA (docosahexaenoic acid) fatty acids, can regulate tight junction functions and serve as precursors for the synthesis of anti-inflammatory eicosanoids, which may help reduce inflammation in the intestine and maintain intestinal integrity. Omega-3 PUFAs can modify the intestinal microbiota through the production and secretion of intestinal alkaline phosphatase, reducing the number of LPS-producing bacteria and consequently decreasing metabolic endotoxemia, which is linked to improved intestinal barrier function [165,166].

In the study by Smith et al. (2014) [156], the effect of multivitamin supplementation with and without omega-3 (from 1 mL of fish oil) was evaluated, with an assessment of intestinal permeability done at 12 and 24 weeks, among children aged 12 to 35 weeks. Although micronutrient supplementation with or without fish oil showed improvement in the L:M ratio after 12 and 24 weeks, no significant difference in the L:M ratio was found between the intervention group and the control group within the evaluated period. Van Der Merwe et al. (2013) [157] also conducted a study on fish oil supplementation, at a dose of 2 mL, among children aged 3 months (approximately 13 weeks), and found no significant difference in the reduction of the L:M ratio compared to the control group. The results of these studies are similar in terms of the lack of treatment effect from multivitamin supplementation with nutrients and/or omega-3 on intestinal integrity.

Each nutrient has its specific functions in maintaining intestinal health, although they share similar characteristics in their actions. Especially, they play a crucial role in regulating and differentiating components of the immune system, as well as in the expression of tight junction proteins, which are essential for preserving intestinal membrane selectivity. Alteration of intestinal permeability is one of the underlying causes of childhood undernutrition, and in this regard, modifiable factors such as diet should be established as a therapeutic alternative for recovering intestinal barrier function in children worldwide.

In the study conducted by Hendrixson et al. (2022) [148], involving children aged 6 to 59 months with severe undernutrition in Sierra Leone, an intervention with a new ready-to-use therapeutic food made from oats (o-RUTF) was evaluated compared to s-RUTF (without cereals in the composition) among children with severe undernutrition. The results did not show a significant difference in %lactulose between the interventions. Agapova et al. (2018) [149] evaluated the inclusion of two types of beans (common or cowpea) in the feeding of children between 12 and 33 months in a rural area of Malawi. In this study, no significant differences were observed between interventions and control for the improvement of the z-score from length to age. There was a decrease in %lactulose for the common bean intervention group, but not for the lactulose:mannitol test. In both studies, the percentage of lactulose excreted (%L) < 0.20 was adopted as a reference for normality. Rabbani et al. (2004) [150] demonstrated that both offering a diet with green bananas or pectin improved intestinal permeability in children aged 5 to 12 months with persistent diarrhea in Bangladesh, observed through the reduction in lactulose and mannitol recovery, and L:M ratio.

Studies evaluating the influence of nutrient supplementation on the recovery of intestinal barrier function have also been described. Glutamine supplementation presents differences in its effect on intestinal permeability. In the study by Van Den Berg et al. (2006) [151] with infants <34 weeks gestational age and very low birth weight, receiving enteral supplementation of glutamine administered in escalating doses up to a maximum of 0.3 g/kg/day of glutamine (starting with a dose of 0.05 g/kg/day), a similar response was observed in both the glutamine-supplemented group and the control group. Unlike the previous study, Sevastiadou et al. (2011) [152] demonstrated that 0.3 g/kg/day of glutamine in children < 32 weeks gestational age had a beneficial effect on intestinal integrity, as evidenced by a decrease in lactulose recovery, as well as a reduction in the lactulose:mannitol (L:M) ratio over the evaluated study period, compared to the control group.

In the study by Lima et al. (2014) [145], conducted with children under 9 years old from an endemic region of enteric diseases, supplementation with glutamine (16 g), combined with zinc (40 mg) and vitamin A (100,000 or 200,000 IU for those over 12 months), promoted improvements in height-for-age, weight-for-age, weight-for-height z-scores, as well as intestinal permeability, assessed through the recovery of %lactulose and the L:M ratio. Although vitamin A has well-recognized benefits in preventing diarrhea, in the study by Rollins et al. (2000) [153], its direct effect on improving intestinal permeability from supplementation of 60 mg of vitamin A for 3 days did not significantly improve early clinical or biochemical recovery from severe diarrhea in children aged 6 to 60 months, nor the levels of the L:M ratio.

Wessells et al. (2013) [123] conducted an intervention with zinc using two vehicles, a dispersible tablet or liquid supplement containing 5 mg of zinc for 21 days with children aged 6 to 23 months. In this study, two cutoff values were used for changes in the L:M ratio: ≥0.07 or ≥0.03. The prevalence of altered (increased) intestinal permeability was 25.5% and 75.5%, respectively. Long et al. (2019) [154] compared the provision of a multivitamin with minerals, differing only in the zinc concentration: 0 mg, 5 mg, 10 mg, or 15 mg. In this study, L:M ratio values ≥ 0.09 were considered altered, and from this cutoff point, children were divided into groups with low or high L:M ratios. Zinc supplementation did not demonstrate an improvement in intestinal permeability (L:M ratio) in either of the two studies mentioned. Hossain et al. (2010) [155] evaluated the supplementation of multivitamins and minerals, containing vitamins A, B-complex, C, and D, along with calcium, zinc, and iron, combined or not with infant and parental psychosocial stimulation. There were no significant effects of dietary supplementation, combined or not, on changes in urinary clearance of lactulose, mannitol, or on the recovery of L:M, considering altered L:M values ≥ 0.07 in children aged 6 to 24 months.

The studies do not show homogeneity regarding dosage, nutrient vehicle, and treatment duration, which may help explain the differences between positive and negative results of supplementation on improving intestinal integrity in the evaluated studies. Additionally, most studies investigating the association of intestinal permeability in child development and combating undernutrition are indexed to African countries. Therefore, demographic characteristics should also be considered. Apparently, a higher dosage—adjusted by weight and age—seems to be more effective in detecting changes in intestinal permeability in children with compromised nutritional status, especially for interventions with glutamine and vitamin A.

In addition to ensuring the restoration of nutritional status in children suffering from malabsorption in the small intestine, nutrients are also essential for promoting the recovery of intestinal health, as they participate in vital physiological processes by providing the basic elements necessary for proper growth, development, and functioning of the body. In this regard, the specific role of nutrients in intestinal health has been explored.

**Figure 4:**
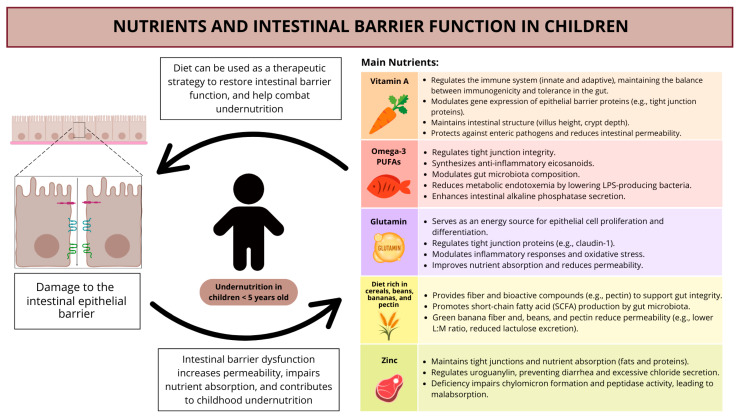
Nutrients and intestinal barrier function in childhood undernutrition.

Based on the findings presented, it is essential to distinguish and discuss two complementary perspectives, nutrients and intestinal barrier function, both as contributing factors to childhood undernutrition and as potential therapeutic targets. Intestinal barrier dysfunction, characterized by increased permeability, impairs nutrient absorption, directly contributing to undernutrition. On the other hand, interventions aimed at restoring intestinal barrier integrity can improve absorption, reduce inflammatory processes, and thus promote nutritional recovery. Similarly, deficiencies in nutrients such as vitamin A, zinc, glutamine, and omega-3 are associated with intestinal dysfunction, while their supplementation can have therapeutic effects by promoting epithelial repair, immune modulation, and maintenance of tight junction integrity. Therefore, understanding these two elements both as causes and as therapeutic targets offers a more integrated and effective approach to addressing childhood undernutrition. Figure 4 summarizes the key aspects of the relationship between nutrients and intestinal barrier function.

## 8. Conclusions

This review demonstrated that enteric infections and environmental enteropathy, along with nutritional deficiencies, are closely associated with childhood undernutrition. Recurrent immune insults and impaired nutrient absorption appear to be the main drivers of growth faltering. Campylobacter, EAEC, and Giardia were identified as key pathogens linked to growth deficits, with EAEC being the most prevalent in co-infections. Measuring increased intestinal permeability remains the primary approach to assess damage to the intestinal epithelial barrier; however, further research is needed to identify and validate more specific and accessible biomarkers for early diagnosis and monitoring.

Moreover, nutrients such as glutamine, vitamin A, zinc, and omega-3 fatty acids play critical roles in maintaining intestinal integrity, modulating inflammation, and supporting epithelial barrier function. Despite their potential, intervention outcomes vary widely depending on dosage, method of administration, and population characteristics. Therefore, future studies should focus on optimizing nutrient-based therapies through personalized approaches, evaluating long-term outcomes of nutritional interventions on gut health and growth, and exploring the role of the gut microbiome in modulating susceptibility to enteric infections and their impact on undernutrition. Such investigations are essential to develop more precise and effective strategies for promoting child health in vulnerable populations.

## Figures and Tables

**Table 1:** Main findings of studies on intestinal infections and diarrhea in children.

Study/Setting	Population	Major Pathogens	Association with Severity/Outcomes	Relevant Observations	References
GEMS—Global Enteric Multicenter Study (Africa and Asia)	9439 children <5 years old	Rotavirus, *Cryptosporidium*, ETEC (ST), *Shigella*, typical EPEC	Moderate to severe diarrhea; Mortality in <2 years	ETEC (ST) and typical EPEC linked to deaths in children < 11 months; *Cryptosporidium* associated with deaths between 12–23 months	[67]
MAL-ED (8 low-income countries: Bangladesh, Brazil, India, Nepal, Pakistan, Peru, South Africa, Tanzania)	1715 children <2 years, 6625 surveillance samples	*Shigella*, sapovirus, rotavirus, adenovirus 40/41, ETEC, norovirus, astrovirus, *Campylobacter jejuni* or *C. coli*, *Cryptosporidium*, typical EPEC, EAEC	*Campylobacter*, EAEC, astrovirus, and *Giardia* associated with severe infections and growth failure; *Giardia* related to persistent infection in the first 6 months of age and growth failure	Viral infections more frequent than bacterial/parasitic in first 2 years; EAEC was more prevalent (94.8%) even without causing diarrhea; *Campylobacter* related to markers of intestinal and systemic inflammation and severe intestinal damage; sapovirus more prevalent in places with anti-rotavirus vaccination, and high probability of co-infection	[44,60,72,79,92,99]
Rotasiil vaccine clinical trial (Niger)	Children <2 years, 1729 episodes	*Shigella*, *Cryptosporidium*, rotavirus, ST-ETEC	*Cryptosporidium* leading cause of severe diarrhea and hospitalization	60.5% of severe shigellosis cases occurred between 12–23 months	[47]
Study in Shanghai (2015–2018)	2692 children with acute diarrhea	Rotavirus (16%), Norovirus (15.5%), NTS (10.3%), EPEC (6.5%), EAEC (6.2%)	Predominance of viral infections; NTS was the most frequently isolated bacterium	Bacterial diagnosis was by culture, possibly underestimating fastidious pathogens	[48]
Brazilian semiarid cross-sectional study	1200 children 2–36 months from six cites	Norovirus GII, Adenovirus, typical EPEC, ETEC LT and ST, rotavirus, STEC, EAEC, and Giardia	Norovirus, adenovirus, rotavirus, STEC, Giardia spp. and EAEC were predictive pathogens for diarrhea	Norovirus linked to poor anthropometric outcomes, co-infection (EAEC and *Shigella*), and respiratory symptoms; EAEC was associated with high diarrhea severity score, and co-infection with rotavirus	[49,50,61]
Peru birth cohort	345 children 0–24 months	ETEC	High burden in neonates and increased infections with age	High recurrence of ST-ETEC infections linked to stunting and wasting post-diarrhea	[75]
Bangladesh child study	1050 stunted and at risk of stunting children	ETEC, EPEC, EAEC, *Shigella*/EIEC, STEC	Growth faltering and inflammation	EAEC and ETEC influences gut health; EPEC is associated with linear growth and underweight	[76]
Bangladesh surveillance study	Children <5 years	ETEC	Common co-infection in diarrheal cases	High prevalence of ETEC co-infections with rotavirus in children	[98]
Co-infection studies (Sub-Saharan Africa and MAL-ED)	Children with diarrhea	No single etiology	59% of samples had co-infections; most with 2 (53%) to 3 (22%) pathogens	Co-infection increases risk of persistent diarrhea, although persistence and severity were not assessed	[97]
Co-infection studies (Sub-Saharan Africa and MAL-ED)	Children without diarrhea	EAEC + Campylobacter, ETEC (LT), *Cryptosporidium*, atypical EPEC	Co-infections associated with increased intestinal inflammation and low weight/height and weight/age	Campylobacter was the pathogen most correlated with EAEC; co-infections with EAEC increase intestinal inflammation and reduce weight/height or age	[80]

**Table 2:** Environmental Enteropathy: etiology, intestinal and systemic changes, diagnostic biomarkers, and animal models.

Category	Population/Model	Finding	Evidence	Observations	References
Etiology	MAL-ED cohort	Enteric infections (Norovirus, Campylobacter, LT-ETEC, *Shigella*, EAEC and co-pathogens)	Associated with growth retardation	Samples with and without diarrhea	[9,44,80]
	Children 9 to 15 months, MAL-ED cohort	Low-fiber diet	Increased EE scores	In children during complementary feeding	[140]
	Children aged 6–23 months in Burkina Faso and <5 years in Sub-Saharan Africa and South Asia	Zinc deficiency	Stunted children with EE	—	[123,124]
Intestinal changes	Upper GI tract of 90 two-year-old children, Bangladesh birth cohort	Quantitative anomaly (>10^5^ CFU/mL) in small intestine	Associated with growth deficits and intestinal inflammation	Changes are independent of recent or frequent diarrhea; no increased intestinal permeability or systemic inflammation	[92]
	Duodenal biopsy transcriptome (3 EE/malnutrition cohorts)	↑ IL-17, ↑ JAK-STAT, ↑ cytokine receptors, ↓ detox/antioxidant capacity	Immune dysfunction and reduced detox capacity	Consistent across study sites	[26]
	Small intestine biopsies, children 0.5–3 years, The Gambia	Villous atrophy, crypt hyperplasia	Common in EE	Compared to children from privileged settings (Europe, Brazil)	[102]
	Clinical studies with children aged 2–16 months	Increased permeability, tight junction alterations	Intestinal epithelial barrier dysfunction	Permeability linked to reduced linear growth and ↑ claudin-4	[103,106]
	Children 0.5–5 years (Gambia, Mexico, Turkey), malnourished elderly	↑ B and T cells, ↑ IFN-γ, ↓ IL-10	Intestinal/systemic immune dysregulation	Lamina propria and blood	[102,107,108,109]
Biomarkers	Serum and stool	↑ A1AT, ↑ Reg-1, ↑ zonulin, ↑ L:M, ↑ LPS, ↑ MPO, ↑ LCN-2, ↑ CRP, ↓ IGF-1	Associated with intestinal inflammation/dysfunction	Not always mutually correlated	[5,100,111,112]
Animal models	Protein-deficient mice/piglets	↑ L:M, ↓ villus height, ↑ MPO, ↑ LCN-2, ↑ TLR2/TLR4, ↓ intraepithelial/lamina propria lymphocytes	Intestinal barrier dysfunction, inflammation, villous atrophy, ↓ CD4+ T cells	No crypt hyperplasia; differs from EE	[113,114,115,116,120,121,122,140]
	Zinc-deficient mice	Impaired Paneth cell function	Only partial EE signs with infection	Alone, does not impact growth or gut morphology	[125]
	Mice on protein/energy/zinc-deficient diets	↓ Villus height, ↑ crypt depth, ↑ permeability, ↓ growth	EE features with chronic injury	No clear histological damage; no assessment of inflammation/bacterial translocation	[31]
	Deficient mice/piglets + infections (EAEC, ETEC, Campylobacter, etc.)	↑ Weight loss, ↑ villus height, ↑ crypt depth (*Cryptosporidium*), ↑ permeability (*E. coli*/Giardia), ↑ inflammation (EAEC, Campylobacter)	Greater inflammation and gut damage	Partially mimic EE; usually no crypt hyperplasia; models need microbiota depletion	[113,115,122,128,129,130,131,132,133,134]
	Gnotobiotic piglets with EE child microbiota	↑ Rotavirus diarrhea	Does not fully reproduce EE; no histological changes	Worsens rotavirus diarrhea	[136]
Other contributing factors	Various studies	Environmental toxins (exposome)	Contribute to EE pathogenesis	May explain limits of animal models	[137,138,139]
